# Epidemiological aspects of rotavirus and adenovirus in hospitalized children with diarrhea: a 5-year survey in Beijing

**DOI:** 10.1186/s12879-016-1829-z

**Published:** 2016-09-23

**Authors:** Liying Liu, Yuan Qian, You Zhang, Linqing Zhao, Liping Jia, Huijing Dong

**Affiliations:** laboratory of Virology, Beijing Key Laboratory of Etiology of Viral Diseases in Children, Capital Institute of Pediatrics, Beijing, 100020 China

**Keywords:** Diarrhea, Gastroenteritis, Rotavirus, Adenovirus, Children, Hospitalization

## Abstract

**Background:**

Diarrhea caused by viruses is a global problem among young children. We investigated two of the most important agents, rotavirus and adenovirus, to provide epidemiological evidence for a better understanding of their role among children with acute diarrhea.

**Methods:**

A total of 3147 hospitalized children were enrolled in the study during 2010 ~ 2014. Antigen testing for rotavirus and DNA testing for adenovirus were performed on stool specimens collected from participants.

**Results:**

There were 1985 cases of community-acquired diarrhea (CAD) and 1162 cases of hospital-acquired diarrhea (HAD). A total of 692 cases (22.0 %) were positive for rotavirus. Rotavirus was detected in more children with HAD than in those with CAD (24.6 %; 286/1162 vs. 20.5 %; 406/1985). A total of 324 cases (10.3 %) were adenovirus positive. There was a significant difference between the CAD group and HAD group (9.5 %; 188/1985 vs. 11.7 %; 136/1162: *χ*^2^ = 3.957, *p* = 0.047). Co-infection was found in only 35 children (1.11 %), and the co-infection rate was similar between the CAD and HAD groups (*χ*^2^ = 1.174, *p* = 0.279). There was no association between sex and the detection rate of these viruses. The positive rate was significantly different for rotavirus among CAD cases (*χ*^2^ = 27.979, *p* < 0.001) and for adenovirus (*χ*^2^ = 34.362, *p* < 0.001) in the five age groups. Compared with the other four age groups (15.8–19.8 %), the prevalence of rotaviruses was highest among children aged 12–24 months (28.6 %). Adenovirus was detected in 3.6 % of neonates compared with 5.8 % of infants from 1 to 6 months old; this increased to 12.0–13.8 % in children over 6 months of age. In HAD cases, age differences were not found for rotavirus and adenovirus. Seasonal variation of rotavirus was observed, with peaks in November and December and with through in July and August; however, no clear seasonal pattern was found for adenovirus.

**Conclusion:**

Detection rates for rotavirus and adenovirus were significantly higher in children with HAD than those with CAD, but co-infection was very low. A high prevalence of rotavirus was identified in neonates with diarrhea. Vaccination for rotavirus gastroenteritis should be considered in neonates.

**Electronic supplementary material:**

The online version of this article (doi:10.1186/s12879-016-1829-z) contains supplementary material, which is available to authorized users.

## Background

Diarrhea remains the leading infectious cause of death in children under the age of 5 years. The global burden of incidence and severe disease for diarrhea is very high, especially in developing countries [1]. Rotavirus is the main cause of severe diarrhea in children below 5 years of age worldwide. The virus is associated with considerable hospitalizations and deaths among children and is responsible for large healthcare expenditures [2]. The most common virus strains are G1P [3], G2P [4], G3P [3], and G9P [3]. Each year, rotavirus causes an estimated 2 million hospitalizations and 450,000 deaths among children globally, with the majority of deaths occurring in developing countries of Asia and Africa [4, 5]. A total expenditure of USD 365 million to treat rotavirus-associated gastroenteritis has been reported in China [2]. Safe and effective rotavirus vaccines could substantially reduce the burden of disease. The World Health Organization has recommended the use of rotavirus vaccines in routine immunization programs worldwide [6].

Adenovirus is another important etiological agent of serious gastroenteritis among infants and young children. Many serotypes can be detected in children with diarrhea, such as Ad41, Ad3, Ad7, Ad31, and others. A strong association between adenovirus infection and intussusception has been identified in hospitals [7]. In recent years, adenovirus has become an increasingly significant pathogen in children following bone marrow transplantation. Infection with adenovirus can cause severe disease, increased mortality, and prolonged length of hospital stay [8].

No effective treatments have been developed for viral gastroenteritis; therefore, regional and local epidemiological information on rotavirus and adenovirus infection is important for healthcare practitioners and officials to develop suitable vaccines and implement infection control measures [3]. Numerous studies have identified causative viral agents for hospitalized patients and outpatients with community-acquired diarrhea (CAD). However, there are few data for children who develop diarrhea symptoms over 48 h after hospital admission (hospital-acquired diarrhea, HAD). To describe the epidemiological and clinical features of rotavirus and adenovirus among children with CAD and with HAD, we conducted a 5-year study that included virus testing of fecal specimens collected from hospitalized children under 5 years of age with acute diarrhea, from 2010 to 2014 in Beijing, China.

## Methods

### Study setting

The study was performed at the Affiliated Children’s Hospital of the Capital Institute of Pediatrics in Beijing. This hospital has 410 inpatient beds and serves about 1.5 million outpatients and 13,000 inpatients; approximately 2700 surgeries are performed annually.

### Specimen collection

Acute diarrhea is defined as three or more loose or looser-than-normal stools within a 24-h period. Changes present in stool include watery, mucoid, or pasty consistency, with no pus and blood. In this study, fecal specimens were excluded if red blood cells were present or white blood cell counts were over 5 white blood cells/high power field by microscopic examination.

Children who developed diarrhea symptoms within 48 h of admission to the hospital were considered to have community-acquired diarrhea (CAD). If symptoms occurred 48 h or more after hospital admission, then hospital-acquired diarrhea (HAD) was assumed.

Samples were categorized into five groups according to patient age: neonate (<1 month), approximately 6 months (≥1 month and <6 months), approximately 12 months (≥6 months and <12 months), approximately 24 months (≥12 months and <24 months), and approximately 60 months (≥24 months and <60 months).

From January 1, 2010 to December 31, 2014, stool samples were collected from hospitalized children with acute diarrhea. All specimens were kept frozen until testing.

### Laboratory virus testing

Stool samples were screened using the Rotascreen® Dipstick (Beijing WanTai Biological Pharmacy Enterprise Co. Ltd., Beijing, China) and polymerase chain reaction (PCR), to test for the presence of rotavirus and adenovirus, respectively. Rotavirus testing was carried out according to the manufacturer’s instructions, using the immune colloidal gold technique. For adenovirus, nucleic acid was extracted from fecal specimens using DNAzol (MRC, Inc., Cincinnati, OH, USA) in our modified method that has been published previously [9]. Adenovirus types were assayed by conventional PCR using universal primers [10, 11].

### Data analysis

Medical records for all enrolled study participants were reviewed and information such as sex, age, date of sample collection, and admitting diagnosis was collected. The chi-square (*χ*2) test and *t*-test were used for statistical analysis and data were analyzed using IBM SPSS Statistics for Windows, Version 19.0 (IBM Corp., Armonk, NY USA). Significance was set to *p* < 0.05.

## Results

### Diarrhea classification

In this study, we enrolled 3147 hospitalized children with diarrhea; 1985 cases (63.1 %) were defined as CAD and 1162 cases (36.9 %) were considered HAD.

### Rotavirus and adenovirus detection rates

A total of 692 children (22.0 %, 692/3147) were positive for rotavirus, including 406 cases (20.5 %, 406/1985) with CAD and 286 (24.6 %, 286/1162) with HAD. There was a significant difference between the CAD group and HAD group for rotavirus (*χ*^2^ = 7.392, *p* = 0.007). A total of 324 cases (10.3 %, 324/3147) were positive for adenovirus. The difference for adenovirus was yet significant between infants with CAD and with HAD (*χ*2 = 3.957, *p* = 0.047), and there was a higher prevalence (11.7 %, 136/1162) found among children with HAD compared with CAD (9.5 %, 188/1985). Co-infection was found in only 35 children (1.1 %). No difference in the co-infection rate was found between the CAD (1.0 %, 19/1985) and HAD groups (1.4 %, 16/1162).

A total of 461 children hospitalized for acute diarrhea were included in the CAD group. Of these, 115 (25.0 %) tested positive for rotavirus and 39 (8.5 %) were positive for adenovirus; co-infection was found in only eight children (1.7 %). The average length of hospital stay for these participants was 7.4 days, and no difference in length of hospital stay was found among rotavirus (7.0 days), adenovirus (8.4 days), and co-infection (8.3 days).

### Rotavirus and adenovirus detection rates according to sex

Fecal specimens were collected from 1979 male and 1168 female subjects. In the CAD group, 20.2 % of male subjects and 20.9 % of female subjects tested positive for rotavirus. The positive rate for adenovirus was 9.9 % of male subjects and 8.8 % of female subjects. There were 14 (1.2 %) co-infection cases among male subjects and 5 (0.7 %) among female subjects. Among the HAD group, rotavirus, adenovirus, and co-infection were identified in 24.2, 13.0, and 1.7 % of male subjects, respectively, compared with 25.4, 9.1, and 0.8 % of female subjects. As shown in Table 1, there was no association between the sex of participants and the detection rates of rotavirus, adenovirus, and co-infection in both the CAD and HAD groups.

**Table 1:** Sex and age distribution of children with CAD and HAD in Beijing, China, 2010–2014

Group	CAD				HAD			
	Number	Ra	Ad	Co	Number	Ra	Ad	Co
Male	1211	244	120	14	768	186	100	13
Female	774	162	68	5	395	100	36	3
*χ* ^2^		0.177	0.695	1.296		0.189	3.801	1.663
*p*		0.674	0.404	0.255		0.663	0.051	0.197
Total	1985	406	188	19	1162	286	136	16
Neonate	247	39	9	0	68	18	3	0
~6 months	607	104	35	3	312	79	36	5
~12 months	414	78	57	5	268	67	38	6
~24 months	490	140	59	7	325	87	37	4
~60 month	227	45	28	4	189	35	22	1
*χ* ^2^		27.979	34.362	6.7334		4.830	5.129	3.584
*p*		**<0.001**	**<0.001**	0.151		0.305	0.274	0.465

*Abbreviations*: *CAD* community-acquired diarrhea, *HAD* hospital-acquired diarrhea, *Ra* rotavirus, *Ad* adenovirus, *Co* co-infection  Note: Significance was set to *p* < 0.05

### Rotavirus and adenovirus detection rates by age group

The median age of study participants was 9.0 months (range: 1 day to 60 months). The median age of children with CAD (7.73 months, range: 3 days to 60 months) differed from that of participants with HAD (10.45 months, range: 1 day to 60 months). Children with HAD were older (*t* = −5.83, *p* < 0.010). However, there was no difference between the CAD and HAD groups for the median age of rotavirus-positive cases (*t* = 0.12, *p* = 0.500) or those positive for adenovirus (*t* = −0.35, *p* = 0.360).

**Fig. 1:**
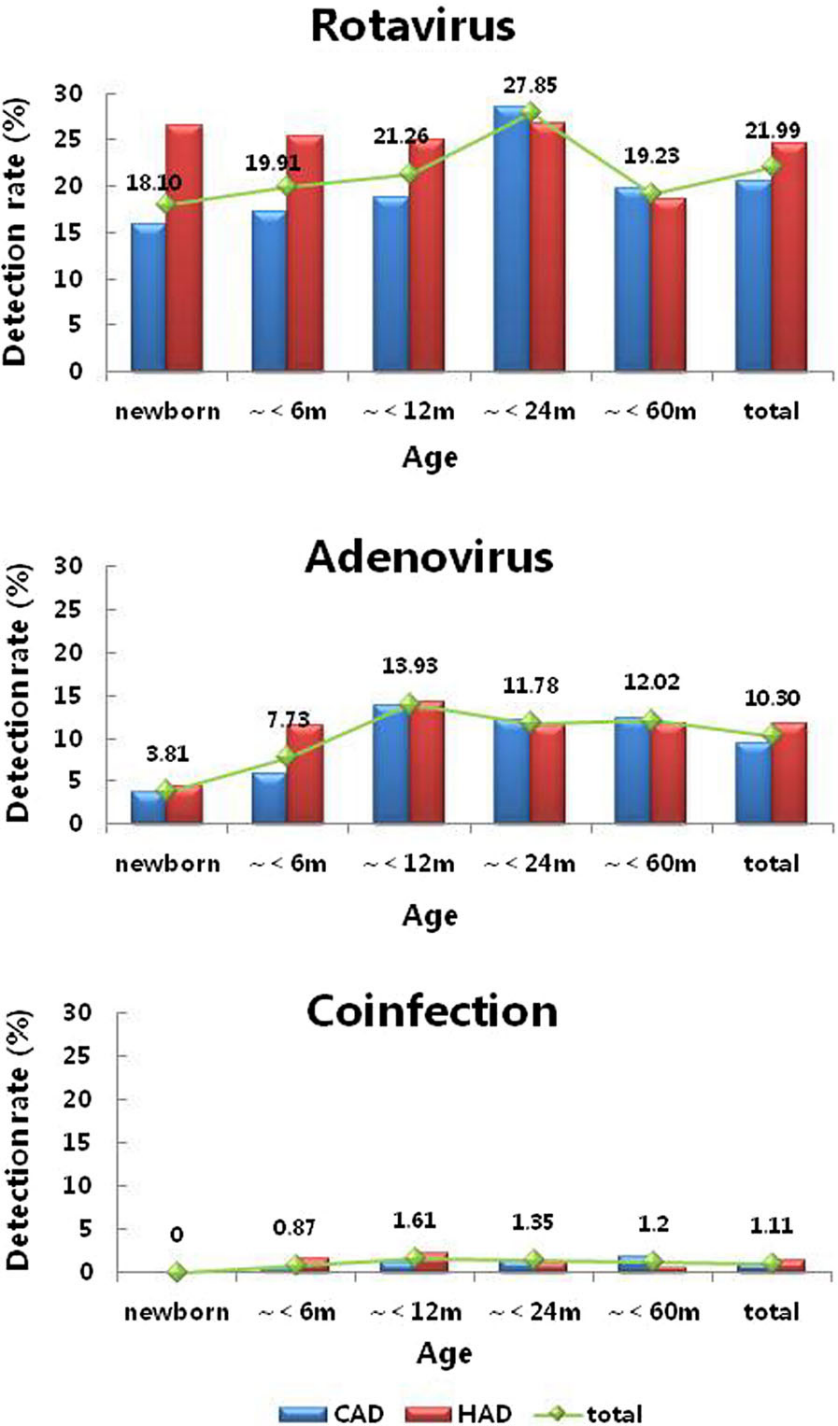
Age distribution of rotavirus and adenovirus detection rates in children with CAD and HAD. CAD: community-acquired diarrhea; HAD: hospital-acquired diarrhea. Age ranges for each age group are indicated in parentheses as follows: neonates (<1 month), ~6 months (≥1 to <6 months), ~12 months (≥6 to <12 months), ~24 months (≥12 to <24 months), ~60 months (≥24 to <60 months)

As shown in Table 1 and Fig. 1, the positive rate in the CAD group was significantly different for rotavirus (*χ*^2^ = 27.979, *p <* 0.001) and adenovirus (*χ*^2^ = 34.362, *p <* 0.001) among the five age groups in the study. The prevalence was highest among children from 12 to 24 months old for rotavirus (28.6 %); this was similar among the other four age groups (15.8–19.8 %). Adenovirus was detected in 3.6 % of neonates compared with 5.8 % of infants from 1 to 6 months old and increased to 12.0–13.8 % in children over 6 months. In the HAD group, no differences were found for rotavirus or adenovirus among the five age groups. However, compared with the CAD group, a notable increase could be seen for adenovirus in children with HAD from 1 to 6 months and for rotavirus in infants under 6 months old. There were no differences in the detection of both viruses between the CAD and HAD groups.

### Seasonality of rotavirus and adenovirus

**Fig. 2:**
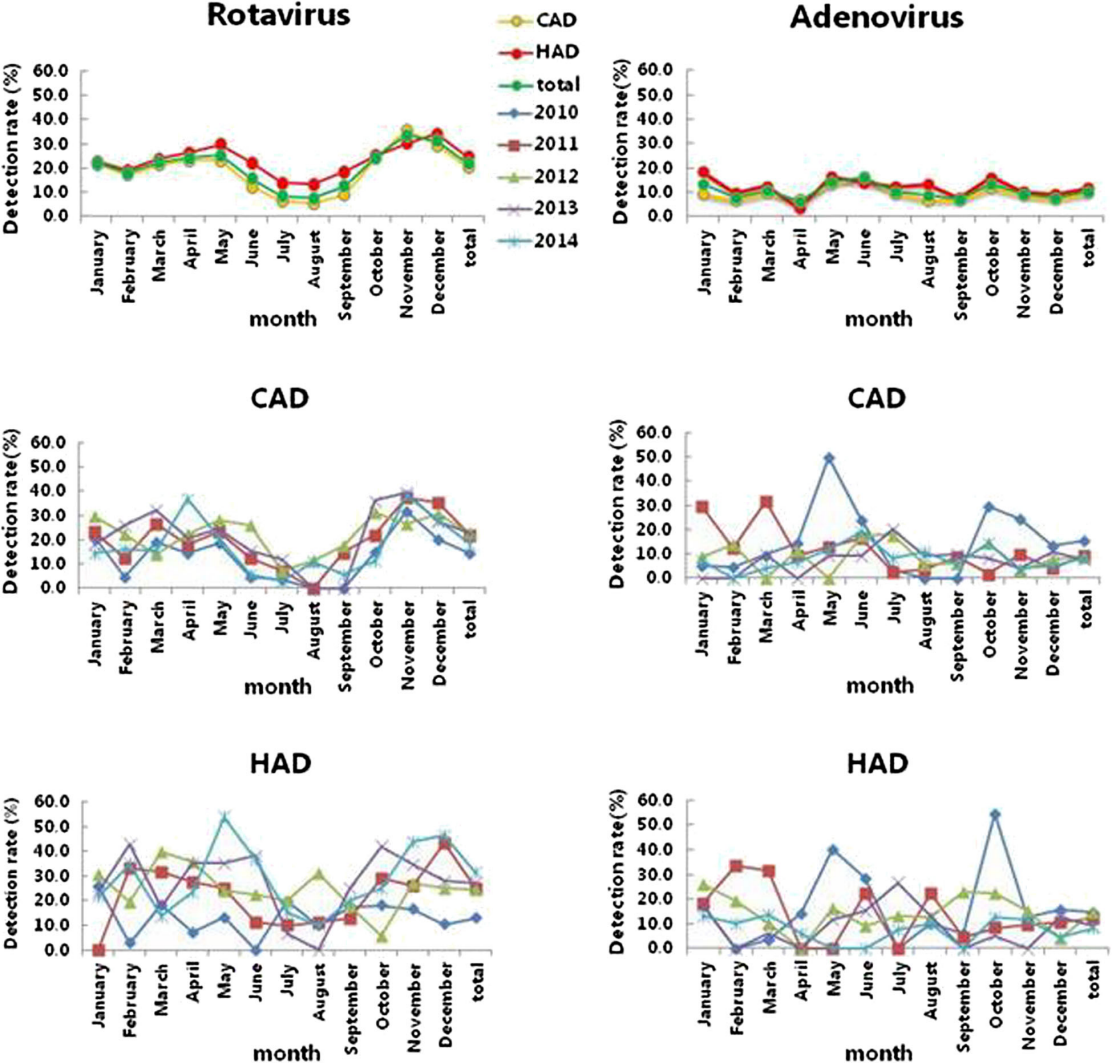
Monthly distribution of rotavirus and adenovirus detection in children with CAD and HAD. CAD: community-acquired diarrhea; HAD: hospital-acquired diarrhea

Over the 5-year study period, seasonal variation was observed for rotavirus among hospitalized children with diarrhea. Peaks in rotavirus prevalence occurred in November (33.7 %) and December (31.3 %) and arrived at through in July (8.3 %) and August (7.7 %). However, this seasonal pattern was seen each year only for the CAD group. There was large variation in the monthly detection rate of rotavirus in each of the 5 years among children in the HAD group. Adenovirus was detected throughout the year, with no clear seasonal pattern found among children with CAD and HAD. Monthly distribution of adenovirus-positive cases varied widely each year during the study period. Such seasonal patterns of infection have been demonstrated in general pediatric populations, as shown in Fig. 2.

## Discussion

Diarrhea is a major cause of childhood morbidity and mortality worldwide. Rotavirus and adenovirus are recognized as common etiologies of acute diarrhea. In our 5-year study, detection rates of 22.0 % for rotavirus and 10.3 % for adenovirus provided evidence supporting their importance in infantile diarrhea. These results were found among children hospitalized not only for acute diarrhea but also for reasons such as respiratory diseases, neurological disorders, cardiovascular diseases, renal diseases, and others. According to the time of diarrhea onset, our study participants were categorized as having CAD or HAD; the 48-h symptom-based definition adopted here has been used extensively in many studies [12–14]. Compared with other studies, this research involved a larger cohort size and longer investigation period. Analysis of larger datasets can better clarify the epidemiological characteristics of rotavirus and adenovirus in children with diarrhea and can serve to reduce bias.

Positive rates of rotavirus and adenovirus vary in different areas and countries owing to differences in detection methods and the tested populations. In China, rotavirus usually accounts for 18.4–54.0 % of children with acute diarrhea under the age of 5 years [15, 16]. The rotavirus detection rate in this study was relatively low (22.0 %), perhaps because of the larger cohort size and more children with HAD who were not included in other studies. The highest prevalence was seen among children between 12 and 24 months old, which is similar to several reports [17–19]. Most of the literature considers that the incidence of rotavirus is greatest during the first 2 years of life [16, 20]; the same conclusion was also reached in our study. Newborns were also evaluated in this research. A total of 315 infants under 1 month were enrolled and a high positive rate (18.1 %) was found for rotavirus. However, relevant data are absent in many investigations of children with diarrhea, even though neonates are at an important age for high infection rate and mortality [5]. In this study, the peak incidence season of rotavirus differed by geographical latitude and began from August to September in Guangdong Province [17], November to December in Liaoning Province [21], and in October in Beijing. Reported rates of adenovirus infection in China range from 0.3 to 40.7 % in different studies [21, 22]; this variability may be owing to differences in sample selection, diagnostic methods used (e.g., PCR, enzyme-linked immunosorbent assay, and gold immunochromatographic assay), and others. The detection rate of adenovirus in this study was 10.3 %, which was high among numerous studies because of the use of PCR primers for all of adenovirus types. Ad41 and Ad31 are the most common adenovirus serotypes among hospitalized children with either CAD or HAD, which has been reported in other research by our group [23]. In most published literature, the co-infection rates of rotavirus and adenovirus are very low, which is consistent with the rate found in this work (1.1 %) [16, 17, 24]. Children with HAD require greater protection from viral infection; higher positive rates were found in the HAD group than the CAD group for the two viruses investigated here. Vaccination can prevent more serious illness, avoid longer hospital stays, and reduce healthcare expenditure.

Rotavirus is associated with considerable hospitalizations and deaths among children worldwide, and large annual societal costs for treatment have been reported in many countries [1, 2, 4]. Safe and effective rotavirus vaccines could substantially reduce the burden of disease [2]. To date, rotavirus vaccines are not yet included in routine immunization programs of China; however, some studies, including this research, recommend the introduction of effective rotavirus vaccines [20]. Two live oral rotavirus vaccines are widely available: RotaTeq® (live, oral, pentavalent; Merck & Co, Inc.), which contains five human-bovine reassortant rotavirus strains expressing human serotypes G1, G2, G3, G4, and P1A [3]; and Rotarix™ (live, oral, monovalent; GlaxoSmithKline Biologicals) containing G1P1A [3]. Both vaccines have been shown to be generally safe and effective in preventing rotavirus gastroenteritis in high-, middle-, and low-income countries [25]. G3P [3] and G1P [3] are the most common rotavirus serotypes in China [15, 16, 26]. In the United States, the minimum age is 6 weeks for injection of the above vaccines according to the “Recommended immunization schedule for persons aged 0 through 18 years – United States, 2015”. However, our data suggest that neonates represent a group in which rotavirus vaccination should be considered owing to the high detection rate for this virus in that population. In our study, there was a significant difference in detection rates between rotavirus and adenovirus among children under 6 months old, and especially for neonates (Fig. 1). Maternal antibodies normally protect infants from pathogens for the first 6 months. This coincides with the adenovirus detection rate among the children in our study. The rate of adenovirus positives showed a marked increase in children over 6 months with CAD. Even in the HAD group, there was a lower detection rate (4.4 %) for adenovirus among neonates. This demonstrates that maternal antibodies were effective in protecting against common adenoviral types that cause gastroenteritis in infants younger than 6 months old. However, maternal antibodies did not prevent these children from rotavirus infection. The detection rate of rotavirus in neonates with HAD was high (15.8 %); this was lower than the rate for children aged 12–24 months (28.6 %) but similar to that of other age groups (17.1–20.5 %). The positive rate of rotavirus in neonates was higher in the HAD group (26.5 %); another study reported 25.9 % of neonates tested positive for rotavirus [27]. Our data further support that maternal antibodies are effective in protecting against adenovirus [8] but not rotavirus in infants younger than 6 months old, especially newborns. Rotavirus vaccine is necessary to prevent children from contracting rotavirus-associated diarrhea, and neonates should be considered for immunization against the virus.

## Conclusions

This study presents the epidemiologic and clinical characteristics of rotavirus and adenovirus in hospitalized children with CAD and HAD. A high prevalence of rotavirus was identified in neonates with both CAD and HAD. Our findings will be useful for clinicians and public health workers when considering whether neonates should be included in rotavirus vaccine programs. Further research involving a large number of patients from different institutions and geographic areas is warranted, to confirm these findings.

## Additional file

Additional file 1: Raw data on which this study is based. (XLSX 204 kb)

## Abbreviations

Ad: Adenovirus
CAD: Community-acquired diarrhea
HAD: Hospital-acquired diarrhea

## References

[1] Walker CL, Rudan I, Liu L, Nair H, Theodoratou E, Bhutta ZA, O’Brien KL, Campbell H, Black RE (2013). Global burden of childhood pneumonia and diarrhoea. Lancet (London, England).

[2] Kawai K, O’Brien MA, Goveia MG, Mast TC, El Khoury AC (2012). Burden of rotavirus gastroenteritis and distribution of rotavirus strains in Asia: a systematic review. Vaccine.

[3] Clark B, McKendrick M (2004). A review of viral gastroenteritis. Curr Opin Infect Dis.

[4] Wang H, Liddell CA, Coates MM, Mooney MD, Levitz CE, Schumacher AE, Apfel H, Iannarone M, Phillips B, Lofgren KT (2014). Global, regional, and national levels of neonatal, infant, and under-5 mortality during 1990–2013: a systematic analysis for the Global Burden of Disease Study 2013. Lancet (London, England).

[5] Liu L, Oza S, Hogan D, Perin J, Rudan I, Lawn JE, Cousens S, Mathers C, Black RE (2015). Global, regional, and national causes of child mortality in 2000–13, with projections to inform post-2015 priorities: an updated systematic analysis. Lancet (London, England).

[6] World Health Organization. Meeting of the immunization Strategic Advisory Group of Experts, April 2009--conclusions and recommendations. Wkly Epidemiol Rec. 2009;84(23):220–236. 19499606

[7] Bines JE, Liem NT, Justice FA, Son TN, Kirkwood CD, de Campo M, Barnett P, Bishop RF, Robins-Browne R, Carlin JB (2006). Risk factors for intussusception in infants in Vietnam and Australia: adenovirus implicated, but not rotavirus. J Pediatr.

[8] Walls T, Shankar AG, Shingadia D (2003). Adenovirus: an increasingly important pathogen in paediatric bone marrow transplant patients. Lancet Infect Dis.

[9] Liu L-y ZY, Deng J, Qian Y (2009). Developing of a method for detection and identification of entero-adenovirus in fecal specimens from infantile diarrhea. Chin J Lab Med.

[10] Allard A, Girones R, Juto P, Wadell G (1991). Polymerase chain reaction for detection of adenoviruses in stool samples. J Clin Microbiol.

[11] Allard A, Albinsson B, Wadell G (2001). Rapid typing of human adenoviruses by a general PCR combined with restriction endonuclease analysis. J Clin Microbiol.

[12] Fischer TK, Bresee JS, Glass RI (2004). Rotavirus vaccines and the prevention of hospital-acquired diarrhea in children. Vaccine.

[13] Gleizes O, Desselberger U, Tatochenko V, Rodrigo C, Salman N, Mezner Z, Giaquinto C, Grimprel E (2006). Nosocomial rotavirus infection in European countries: a review of the epidemiology, severity and economic burden of hospital-acquired rotavirus disease. Pediatr Infect Dis J.

[14] Cunliffe NA, Booth JA, Elliot C, Lowe SJ, Sopwith W, Kitchin N, Nakagomi O, Nakagomi T, Hart CA, Regan M (2010). Healthcare-associated viral gastroenteritis among children in a large pediatric hospital, United Kingdom. Emerg Infect Dis.

[15] Zheng-cai DZ-h Yao, Hao Yang, Shuai-feng Zhou, Shi-xiong Hu. Pathogens causing viral diarrhea in children aged ≤ 5 years in Xinhuang, Hunan, 2009–2011. Dis Surveill. 2012;12(27):937–943.

[16] Jin Y, Cheng WX, Yang XM, Jin M, Zhang Q, Xu ZQ, Yu JM, Zhu L, Yang SH, Liu N (2009). Viral agents associated with acute gastroenteritis in children hospitalized with diarrhea in Lanzhou, China. J Clin Virol.

[17] CHEN H, HU T, YAO Y, HUANG Y, XIAO N, LIU X, XIAO Y, CHEN Q, YU S. Etiological and epidemic characterization of viral diarrhea in children under the age of 5 years in Guangzhou City. Chin J Dis Control Prev. 2014;18(4):336–339.

[18] WU J, YAN Y. Analysis on detection results of rotavirus and adenovirus in children with diarrhea. Chinese Journal of Microecology. 2014;26(9):1069–1071.

[19] Li J, ZHOU S, LIU Y, DENG Z, HUANG W, LI D, ZHANG F, YAO Z, YUAN D, LIU F, CHEN Y, ZHANG H. Etiological Study on Viral Diarrhea Among Infants and Young Children in Surveillance Hospitals of Hunan Province from 2009 to 2010. Practical Preventive Medicine. 2012;19(3):337–341.

[20] Duan ZJ, Liu N, Yang SH, Zhang J, Sun LW, Tang JY, Jin Y, Du ZQ, Xu J, Wu QB (2009). Hospital-based surveillance of rotavirus diarrhea in the People’s Republic of China, August 2003-July 2007. J Infect Dis.

[21] AN S, ZHAO Z, GUO J, HAN Y, WANG Z, REN Y, ZHOU B. Epidemiological study on viral diarrhea during 2009–2011 in Liaoning Province. Chin J Infect Dis. 2013;31(3):166–169.

[22] Geng L, GONG S, HE W. Etiology of watery diarrhea in infants in Guang Zhou city, 2003–2006. Guangdong Medical Journal. 2008;29(8):1336–1339.

[23] Liu L, Qian Y, Zhang Y, Deng J, Jia L, Dong H (2014). Adenoviruses associated with acute diarrhea in children in Beijing, China. PLoS One.

[24] Zhao YQI, He S (2011). Analysis of the infants virus diarrhea disease surevillance result in Jilin City between 2007 and 2008. Chin J Lab Diagn.

[25] Armah GE, Sow SO, Breiman RF, Dallas MJ, Tapia MD, Feikin DR, Binka FN, Steele AD, Laserson KF, Ansah NA (2010). Efficacy of pentavalent rotavirus vaccine against severe rotavirus gastroenteritis in infants in developing countries in sub-Saharan Africa: a randomised, double-blind, placebo-controlled trial. Lancet (London, England).

[26] Zhang XE, Li DD, Li X, Yang XD, Cai K, Wang YX, Yang LB, Duan Z (2012). Etiological and epidemiological study on viral diarrhea among children in Changchun. Zhonghua Shi Yan He Lin Chuang Bing Du Xue Za Zhi.

[27] Jia Na-er, Yao Tong, Yu Liang ZBLQ-fLX-h, Du Shuang, Sun He. Rotavirus and adenovirus infections among diarrhea children in Urumqi of China. Chin J Viral Dis*.* 2013;3(06):432–434

